# Treatment outcomes in multidrug resistant tuberculosis-human immunodeficiency virus Co-infected patients on anti-retroviral therapy at Sizwe Tropical Disease Hospital Johannesburg, South Africa

**DOI:** 10.1186/s12879-015-1214-3

**Published:** 2015-10-28

**Authors:** Teye Umanah, Jabulani Ncayiyana, Xavier Padanilam, Peter S. Nyasulu

**Affiliations:** Division of Epidemiology and Biostatistics, School of Public Health, Faculty of Health Sciences, University of the Witwatersrand, Johannesburg, South Africa; Department of Epidemiology, Gillings School of Global Public Health, University of North Carolina at Chapel Hill, Chapel Hill, NC USA; Sizwe Tropical Disease Hospital, Gauteng Department of Health, Sandringham, Johannesburg; Department of Public Health, School of Health Sciences, Monash University, 144 Peter Road, Rumsuig Johannesburg, South Africa

**Keywords:** Mortality, Treatment failure, ‘Timing of ART initiation- before or after commencement of MDR-TB treatment’, MDR-TB/HIV treatment adherence

## Abstract

**Background:**

Multidrug resistant-tuberculosis (MDR-TB) is a threat to global tuberculosis control which is worsened by human immune-deficiency virus (HIV) co-infection. There is however paucity of data on the effects of antiretroviral treatment (ART) before or after starting MDR-TB treatment. This study determined predictors of mortality and treatment failure among HIV co-infected MDR-TB patients on ART.

**Methods:**

A retrospective medical record review of 1200 HIV co-infected MDR-TB patients admitted at Sizwe Tropical Disease Hospital, Johannesburg from 2007 to 2010 was performed. Chi-square test was used to determine treatment outcomes in HIV co-infected MDR-TB patients on ART. Multivariable logistic regression and Poisson models were used to determine predictors of mortality and treatment failure respectively.

**Results:**

Mortality was higher (21.8 % vs. 15.4 %) among patients who started ART before initiating MDR-TB treatment compared with patients initiated on ART after commencing MDR-TB treatment (*p* = 0.013). Factors significantly associated with mortality included: the use of ART before starting MDR-TB treatment (OR 1.65, 95 % CI 1.02–2.73), severely-underweight (OR 3.71, 95 % CI 1.89–7.29) and underweight (OR 2.35, 95 % CI 1.30–4.26), cavities on chest x-rays at baseline (OR 1.76, 95 % CI 1.08–2.94), presence of other opportunistic infections (OR 1.80, 95 % CI 1.10–2.94) and presence of other co-morbidities (OR 2.26, 95 % CI 1.20–4.21). Factors predicting failure were severe anaemia (IRR (OR 4.72, 95 % CI 1.47–15), other co-morbidities (OR 2.39, 95 % CI 1.05–5.43) and modified individualised regimen at baseline (OR 2.15, 95 % CI 0.98–4.71).

**Conclusions:**

High mortality among patients already on ART before initiating MDR-TB treatment is a worrisome development. Management of adverse-events, opportunistic infections and co-morbidities in these patients is important if the protective benefits of being on ART are to be maximized. There is the need to intensify intervention programmes targeted at early identification of MDR-TB, treatment initiation, drug monitoring and increasing adherence among HIV co-infected MDR-TB patients.

## Background

Globally, about 480,000 incident Multidrug-Resistant tuberculosis (MDR-TB) cases were diagnosed among pulmonary TB (PTB) patients in 2013 [1], which is an increase compared to the 450,000 cases reported in 2012 [2]. About 210 000 of the 1.5 million TB deaths globally in 2013 were due to MDR-TB [1]; which is an increase compared to 170,000 deaths of the 1.3 million TB related deaths in 2012 [2]. South Africa is included as one of the countries by the WHO to have over 80 % of the estimated MDR-TB cases worldwide [2].

MDR-TB poses a threat to global TB control programmes [3], and this is aggravated in resource limited and low-income countries due to inadequate availability of prompt diagnostic and treatment measures [4]. The co-infection of MDR-TB and HIV has been described by Wells et al. as the “Perfect storm” [5]. In South Africa the MDR- and XDR-TB epidemic have mainly been driven by the HIV epidemic and inadequate airborne infection control measures [6]. The rapid spread of MDR-TB among HIV-positive patients is due to the impaired ability of the immune system to contain the MDR-TB bacilli in these patients as this applies also to the normal TB bacilli [7]. The probability of developing TB among people living with HIV is 29 times higher compared to the probability of developing TB among HIV-negative people [1].

Diagnosis and treatment of MDR-TB has been quite a challenging venture around the globe especially in the resource limited settings. This is even more difficult among HIV-infected individuals as the classical symptoms of PTB may not be as pronounced as in HIV negative patients. Also HIV positive patients are sometimes smear negative, hence presentations of TB amongst these patients may be over-looked and attributed to the HIV infection. Further difficulties in early diagnosis can be ascribed to delays in results of drug susceptibility testing (the gold standard for diagnosis of MDR-TB) which is sometimes not feasible at baseline for all patients. The adverse events from lifelong treatment of HIV with antiretroviral therapy (ART) coupled with side effects from MDR-TB drugs make the management and outcomes of MDR-TB in co-infected patients very challenging [4].

Research work carried out on timing of ART initiation in patients with pan susceptible TB used ARV-naïve TB patients [8], without looking at the effect of ART initiation before the start of TB treatment. There have been few reported studies looking at the effect of timing of initiation of ART on the outcomes of MDR-TB treatment in a cohort of HIV positive patients [9–11], but none in Sub-Saharan Africa (SSA). These studies did not consider timing of ART based on before or after initiation of MDR-TB treatment. A study done in SSA by Satti H, et al. for both HIV infected and un-infected patients during a sub-group analysis revealed no association between being on ART prior to start of MDR-TB treatment with increased hazard of treatment failure or mortality, although the comparison group was the ART-naïve patients [12].

Hence, data is highly needed in SSA on the effect of timing of ART initiation before or after commencement of MDR-TB treatment on MDR-TB outcomes in HIV positive patients; in order to help evaluate the efficiencies/deficiencies of MDR-TB treatment and ART programmes. Therefore it is essential to ascertain the role timing of introduction of ART plays in the management of patients with MDR-TB and HIV co-infection. Such findings could guide policy change so as to improve MDR-TB treatment among HIV co-infected patients in South Africa. The objectives of this study were to describe the clinical characteristics and outcomes of MDR-TB treatment; and determine the predictors of mortality, and treatment failure in HIV-TB co-infected patients who started ART before and after commencement of MDR-TB treatment.

## Methods

### Study design

This was a retrospective review of medical records of HIV positive MDR-TB adult patients who were registered into the South African National MDR-TB program from 1^st^ January 2007 to 31^st^ December 2010 and receiving treatment at Sizwe Tropical Disease Hospital.

### Study setting and study population

Sizwe Tropical Disease Hospital (STDH) is situated at Edenvale and was established in 1895. In 2002, it became a specialised Provincial centre for the treatment of MDR-TB. In 2006, it was also mandated to treat XDR-TB following the emerging burden of DR-TB in South Africa. The hospital serves a vast majority of referred MDR/XDR-TB patients in Gauteng Province, who are treated as in- and out-patients. The study population comprised all TB patients aged 18 years and older registered for MDR-TB treatment at STDH.

### Study sample

**Fig. 1:**
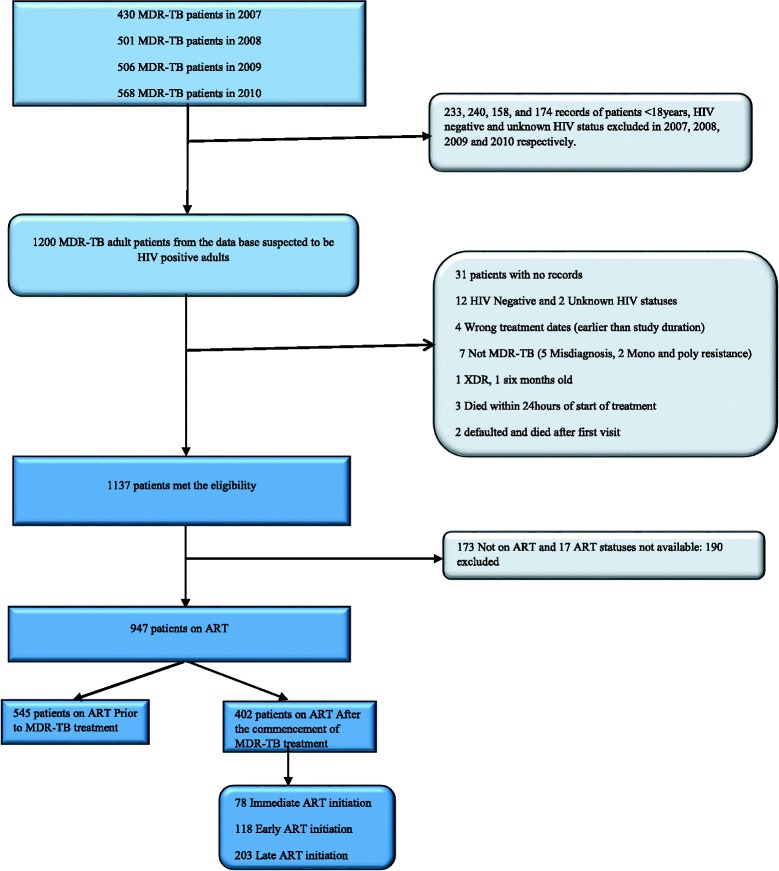
Exclusion and inclusion criteria flowchart of the cohort

A total of 1200 records of adult patients from the database of STDH who were MDR-TB/HIV co-infected were reviewed over a four year period. The following eligibility criteria were applied to ensure that the study sample was correctly selected (Fig. 1). The inclusion criteria included patients on treatment for MDR-TB who were: HIV positive adults aged 18 years and older, with confirmed drug susceptibility test results showing resistance to isoniazid and rifampicin. Patients younger than 18 years, and those who were HIV negative, with unknown HIV status or no HIV test results were excluded from the study. Additionally, those with missing case notes, or whose DST was not confirmatory of MDR-TB, earlier treatment dates before commencement of study, who died within 24 h of commencement of MDR-TB treatment and XDR-TB patients were also excluded from the study.

### Data sources and measurement

Culture positive and confirmed MDR-TB patients using DST or line probe assay (LPA) in all the sub-divisions of the National Health Laboratory Service (NHLS) throughout Gauteng province were referred from their clinics to Sizwe Tropical Disease Hospital. Patients were first screened at Gateway clinic to ensure that results were suggestive or confirmatory of MDR-TB before registration into the National MDR-TB programme. This was followed by a DST for all the first and second-line anti-tuberculous agents. Baseline measurements of weight, height, haemoglobin level, electrolytes, urea and creatinine, thyroid function test, liver function test, CD4 cell count, and audiometry were also done prior to or during treatment.

Patients with unknown HIV status were referred to Thusong clinic (a special clinic in STDH for HIV management) for HIV counselling and testing. An initial rapid test was done and a reactive result necessitated a confirmation with Architect Abbot (IL, USA) Enzyme Linked Immuno-sorbent Assay (ELISA). Newly diagnosed HIV patients were encouraged to initiate ART as soon as possible except where patients were too ill to commence treatment or declined initiation of ART. Prior to 2010, the WHO guidelines for commencement of ART in patients with a confirmed diagnosis of DR-TB was based on a CD4 count of less than 350 cells/mm^3^.[13] The guideline that followed regulated that patients start ART within 2–8 weeks, irrespective of the CD4 cell count [14, 15]. Patients’ ART status was also confirmed by physicians on admission from the referral clinic or by pill inspection. Patients diagnosed to be HIV positive on admission and those already on ART were referred to Thusong clinic for adherence counselling, continuous ARV administration and follow-up on HIV treatment.

The majority of the patients started the standardized MDR-TB regimen which utilises five drugs namely Kanamycin/Amikacin (KM/AM), Moxifloxacin (MFX), Ethionamide (ETO), Terizidone (TDR), Ethambutol (EMB) and/or Pyrazinamide (PZA) taken at least six days a week for at least 6 months during the intensive phase (IP), followed by at least 18 months of MFX/ETO/PZA/TDR after culture conversion for the continuation phase (CP) [14]. The choice for the next regimen was individualized based on follow-up DST results. Some patient’s regimen were modified at baseline (called individualised regimen at baseline in this study) following the advent of pre-existing serious adverse events, co-morbidities such as diabetes, pre-existing psychosis and the presence of pre-XDR strains (resistance to first line anti-TB agents, and to either one of the two key second line injectable or the flouroquinolone drugs, but not classified as XDR-TB). Most of the patients were transferred to their local clinics with DOTS support groups who continued monitoring patients’ treatment after one or two consecutive negative culture results.

Adverse events such as ototoxicity, nephrotoxicity, hepatotoxicity, joint pains, gastro-intestinal symptoms like nausea and vomiting requiring treatment, visual changes and conjunctivitis, psychosis, depression, peripheral neuropathy, hyperuricemia, hypothyroidism and gynaecomastia were reviewed and managed. Patients were evaluated for co-morbidities such as diabetes mellitus (DM), hypertension, cerebrovascular disease, heart failure, cardiomyopathies, and arrhythmias, asthma and chronic obstructive airway disease, chronic kidney disease, chronic liver disease, pancreatic disorders, deep venous thrombosis and cancers. The presence of opportunistic infections (candidiasis, recurrent oral or genital Herpes simplex virus (HSV) infection, Pneumocystis Jiroveci Pneumonia (PJP), Herpes zoster infection, Cryptococcal infection, Cytomegalovirus infection, cervical dysplasia or cancer, Kaposi sarcoma and HIV-associated nephropathy (HIVAN)) were managed and documented by clinicians during treatment and follow-up.

Follow-up included: visits one month after discharge and subsequently 3 monthly with chest X-rays reviewed during each visit until the patient had completed treatment except where patients had other co-morbidities. Upon completion of treatment, patients were followed up every 6 months for 2 years. Outcomes of MDR-TB treatment included cure, completed treatment, defaulted, failed treatment, transferred out, still on treatment and died [14, 16]. “ART prior” to MDR-TB treatment was defined as patients having been on ART before commencing MDR-TB treatment while “ART after” was defined as patients initiated on ARVs after initiating MDR-TB treatment but before ascertainment of MDR-TB treatment outcome.

### Data collection

Part of the data for this study comprised demographic and outcome records from the National TB registers. The medical case files of HIV positive patients were reviewed for socio-demographic and clinical information not recorded on the TB register. Baseline x-rays of the patients were also reviewed. Data were entered into an Excel spreadsheet. Inconsistencies were verified from case records and errors were corrected. Data collection started on the 14th November, 2013 and ended on the 21st February, 2014.

### Data analysis

Data were transferred from the Excel spread sheets into STATA version 12.0 for analysis. The data were cleaned by excluding individual records that did not meet the eligibility criteria (Fig. 1); and missing data were checked for each variable. Pearson’s chi-square test was used to compare categorical variables across the main exposure groups (receiving ART before or after commencing MDR-TB treatment) and Fisher’s exact test was used where there were sparse data of ≤5 in 20 % of the cells. Continuous variables which followed a normal distribution were compared using the student *t*-test and the Wilcoxon Rank-sum test was used for non-normal/skewed data. All analyses were performed after excluding missing data.

Multivariable logistic regression models were used to assess the effect of timing of ART and other predictors of mortality; while multivariable Poisson models were used to determine the predictors of treatment failure due to the low prevalence of failure compared to the total sample size. Potential confounders were adjusted for in the final multivariable models. Odds ratios were calculated for logistic regression models and incidence rate ratios (IRR) for Poisson regression models at 95 % confidence interval and alpha level of <0.05. Twelve patients who started ART one week prior to commencement of MDR-TB treatment were excluded from the multivariable logistic regression analysis to avoid dilutional effect due to insufficient time for occurrence of a significant therapeutic effect of ART among this group of patients.

### Ethical considerations

The study was approved by the University of the Witwatersrand Human Research Ethics Committee (Medical) in 2013. Ethics approval number M130961.

## Results

### Baseline characteristics

A total of 2005 MDR-TB patients were admitted from 1st January, 2007 to 31st December, 2010. Of these, 1200 adult patients had HIV positive status recorded on the database out of which 253 patients were excluded from the study. Of the 253 individuals excluded, 31 had missing case notes, 12 were confirmed HIV negative, 2 had unknown HIV status, 173 were not on ART, while the ART status of 17 of them could not be determined. Additionally, 7 patients were misdiagnosed as MDR-TB (mono/poly resistance), 3 had earlier treatment start dates, 3 died within 24 h of starting treatment, and two defaulted/died after the first visit. One patient with XDR-TB and an infant were also excluded. Therefore 947 patients who were on ART met the eligibility criteria (Fig. 1).

A higher proportion of patients 57.5 % (545) were on ART prior to commencing MDR-TB treatment, whereas 42.5 % (402) commenced ART after initiating MDR-TB treatment. The median duration for initiation of ART was 218 days (IQR = 70–570) for patients on ART prior to commencement of MDR-TB treatment and 58 days (IQR = 32–112) for patients initiated on ART after commencing MDR-TB treatment. Of the 545 patients on ART prior to initiating MDR-TB treatment, 12 were commenced on ART one week before the start of MDR-TB treatment and were further excluded from the final multivariable analysis. Extra-pulmonary (EPTB)/PTB was significantly higher among patients who commenced ART before compared with those who started ART after MDR-TB treatment initiation (20.7 % vs. 13.9 %, (*p* = 0.007)). The median time from the start of MDR-TB treatment to mortality was shorter for patients on ART prior to starting MDR-TB treatment 139 days (IQR = 33–411) compared to 321 (IQR = 105–599) for patients initiated on ART after commencing MDR-TB treatment (Table 1).

**Table 1:** Baseline characteristics of MDR-TB/HIV co-infected adults based on exposure to ART before/after the commencement of MDR-TB treatment

Factors	ART Prior	ART After	*p*-value
	N (%) (*N* = 545)	N (%) (*N* = 402)	
Age	36 (31–43)*	35.5 (31–42)*	0.557^b^
Sex			
Male	264 (48.4)	193 (48.0)	0.896^c^
Female	281 (51.6)	209 (52.0)	
Categories of MDR-TB			
Category I (New)	107 (19.6)	107 (26.6)	0.026*^c^
Category II	438 (80.4)	295 (73.4)	
Pre-treatment smear status			
Negative	166 (30.5)	134 (33.3)	0.595^b^
Positive	317 (58.2)	221 (55.0)	
Not available	62 (11.4)	47 (11.7)	
Site of TB			
Pulmonary	432 (79.3)	346 (86.1)	0.007*^c^
Extra-Pulmonary + Pulmonary	113 (20.7)	56 (13.9)	
BMI categories (Kg/m^2^)			
Severely Underweight (<16)	68 (15.4)	41 (11.4)	0.174^c^
Underweight (≥16–18.49)	130 (29.4)	111 (31.0)	
Normal (≥18.5–24.99)	195 (44.1)	176 (49.2)	
Overweight/ obese (25–29.99)	49 (11.1)	30 (8.4)	
Co-trimoxazole Prophylaxis			
No	21 (4.4)	32 (9.7)	0.003*^c^
Yes	459 (95.6)	297(90.3)	
CD4 cell count (mls/mm^3^)	160.5 (89–270)^a^	156 (66–257)^a^	0.1906^b^
CD4 Categories (mls/mm^3^)			
<150	241 (46.5)	187 (48.1)	0.296^c^
150–349	209 (40.4)	140 (36.0)	
≥350	68 (13.1)	62 (15.9)	
Haemoglobin (g/dl)	11.3 (2.4)*	10.8(2.2)*	0.0012*^d^
Haemoglobin (g/dl)			
Severe Anaemia (<7.0)	24 (4.5)	13 (3.3)	0.009*^c^
Moderate Anaemia (7.0–9.9)	120 (22.3)	123 (31.0)	
Mild Anaemia (10–10.99)	85 (15.8)	70 (17.7)	
Normal (≥11)	308 (57.4)	190 (48.0)	
Duration on ART	218 (70–570)^b^	57.5 (32–111.5)^b^	
Outcome duration or Person time (days)	251 (90–725)^b^	720 (330–945)^b^	0.0286*^b^
Treatment-initiation-delay	11 (5–22)^b^	10 (6–21)^b^	0.921^b^
Infiltrative changes on x-ray			
No	24 (4.5)	11 (2.8)	0.179^c^
Yes	513 (95.5)	385 (97.2)	
Cavitary change on x-ray			
No	315 (58.7)	237 (59.8)	0.715^b^
Yes	222 (41.3)	159 (40.2)	
Fibrotic changes on x-ray			
No	77 (14.3)	58 (14.7)	0.883^c^
Yes	460 (85.7)	337 (85.3)	
Other Opportunistic Infections			
No	399 (73.4)	228 (57.1)	<0.001*^c^
Yes	145 (26.6)	171 (42.9)	
Comorbidity			
No	461 (85.8)	345 (88.0)	0.337^c^
Yes	76 (14.2)	47 (12.0)	
Adverse events			
No	295 (54.8)	239 (61.1)	0.055^c^
Yes	243 (45.2)	152 (38.9)	
Regimen type at baseline			
Standardised	370 (68.5)	295 (75.1)	0.029*^c^
Modified individualised	170 (31.5)	98 (24.9)	

Treatment-initiation-delay: Duration from report of MDR-TB to initiation of treatment

N - Total number in each group; % - Column percentages; Numbers may not add-up to total because of missing variables

Test statistic based on ^b^Rank-sum test, ^c^Chi-square test and ^d^ *t*-test

*Significant *p* < 0.05, Mean and Standard Deviation. ^a^Median and Inter-quartile ranges

### Outcomes of MDR-TB by ART treatment groups

Treatment outcomes were not significantly different between patients on ART before and after the commencement of MDR-TB treatment (*p* = 0.096) (Table 2). When the analysis was restricted to patients who died, the proportion of mortality was higher for those on ART prior to beginning MDR-TB treatment (21.8 % vs. 15.4 %, (*p* = 0.013)) Table 2.

**Table 2:** Outcomes of MDR-TB in HIV co-infected adults based on commencement of ART prior to or after MDR-TB treatment initiation

Factors	ART Prior	ART After	*p*-value
	N (%)	N (%)	
Final Outcomes			
Still on treatment	5 (0.9)	8 (2.0)	0.096
Transferred out	26 (4.8)	18 (4.5)	
Treatment Defaulted	105 (19.3)	101 (25.1)	
Treatment Completed	92 (16.9)	71 (17.7)	
Cure	182 (33.4)	130 (32.3)	
Failure	16 (2.9)	12 (3.0)	
Died	119 (21.8)	62 (15.4)	
Died			
No	426 (78.2)	340 (84.6)	0.013*
Yes	119 (21.8)	62 (15.4)	
Failure			
No	529 (97.1)	390 (97.0)	0.965
Yes	16 (2.9)	12 (3.0)	
Total	545	402	

N - Total number in each group

% - Column percentages

*Significant *p* < 0.05

### Pattern of co-morbidities among HIV co-infected MDR-TB patients

For patients with the presence of other co-morbidities, severe sepsis was the highest 18.1 %, followed by hypertension (11.2 %), and diabetes (9.4 %). The proportion of patients with cerebrovascular diseases, deep venous thrombosis and epilepsy were similar; just over 8.0 % each. Congestive cardiac failure and chronic obstructive airway diseases made up over 13 % of the co-morbidities (Table 3).

**Table 3:** Pattern of co-morbidities among HIV co-infected MDR-TB patients at Sizwe Hospital, Johannesburg

Comorbidities	N (%)
Diabetes	15 (9.4)
Hypertension	18 (11.2)
Congestive heart failure	11 (6.9)
Kidney diseases	12 (7.5)
Cerebrovascular diseases	13 (8.1)
Epilepsy	13 (8.1)
Severe sepsis	29 (18.1)
DVT	14 (8.8)
COADs	10 (6.2)
Chronic liver Disease	3 (1.9)
Cancers	2 (1.3)
Others	20 (12.5)

N: Total number in each group; %: percentage in each category

*DVT* Deep venous thrombosis

*COAD* Chronic obstructive airway diseases

### Predictors of mortality

Patients who received ART prior to commencing MDR-TB treatment were 1.7 times more likely to die compared with those who commenced ART after initiation of MDR-TB treatment (OR 1.65, 95 % CI 1.02–2.73, *p* = 0.050), adjusting for confounders such as BMI, haemoglobin, CD4 cell count and the presence of other opportunistic infections. Compared with normal BMI, those who were severely underweight (OR 3.71, 95 % CI 1.89–7.29, *p* <0.001) and underweight (OR 2.35, 95 % CI 1.30–4.26, *p* = 0.005) were more likely to die. Patients with the presence of cavitary lesions on baseline chest x-ray were 1.8 times more likely to die (OR 1.76, 95 % CI 1.08–2.85, *p* = 0.023) compared with patients without cavitary lesions. The presence of other opportunistic infections was associated with a higher odds of mortality compared with patients without other opportunistic infections (OR 1.80, 95 % CI 1.10–2.94, *p* = 0.019). Compared with patients without co-morbidity, individuals with co-morbidities were 2.3 times more likely to die (OR 2.26 95 % CI 1.20–4.27, *p* = 0.012) (Table 4).

**Table 4:** Predictors of mortality in HIV-positive adults on MDR-TB treatment at Sizwe Tropical Disease Hospital, Johannesburg

	^a^Univariable Analysis		^b^Multivariable Analysis	
Factor	OR (95 % CI)	*p*-value	OR (95 % CI)	*p*-value
ART regimen				
After	1.00		1.00	
Prior	1.49 (1.06–2.09)	0.021*	1.65 (1.00–2.73)	0.050*
Age	1.01 (0.99–1.03)	0.481		
Sex				
Female	1.00		1.00	
Male	1.08 (0.78–1.49)	0.661	1.58 (0.94–2.66)	0.085
Site of TB				
Pulmonary	1.00			
Extra-Pulmonary + Pulmonary	1.40 (0.94–2.09)	0.098		
BMI categories (kg/m^2^)				
Severely Underweight (<16)	4.61 (2.77–7.68)	<0.001*	3.71 (1.89–7.29)	<0.001*
Underweight (≥16–18.49)	2.33 (1.49–3.65)	<0.001*	2.35 (1.30–4.26)	0.005*
Normal (≥18.5–24.99)	1.00		1.00	
Overweight/ obese (25–29.99)	1.20 (0.57–2.51)	0.630	2.30 (0.92–5.71)	0.074
Co-trimoxazole Prophylaxis				
No	1.00		1.00	
Yes	1.33 (0.62–2.89)	0.464	1.30 (0.47–3.54)	0.613
CD4 Categories (mls/mm^3^)				
<150	2.88 (1.62–5.14)	<0.001*	1.56 (0.73–3.38)	0.243
150–349	0.91 (0.48–1.72)	0.770	0.63 (0.28–1.42)	0.266
≥350	1.00		1.00	
Haemoglobin (g/dl)	0.76 (0.70–0.82)	<0.001*	0.90 (0.79–1.02)	0.090
Infiltrative changes on x-ray				
No	1.00			
Yes	0.74 (0.33–1.67)	0.470		
Cavitary changes on x-ray				
No	1.00		1.00	
Yes	1.25 (0.89–1.74)	0.191	1.76 (1.08–2.85)	0.023*
Fibrotic changes on x-ray				
No	1.00			
Yes	1.17 (0.71–1.91)	0.527		
Other Opportunistic Infections				
No	1.00		1.00	
Yes	2.54 (1.82–3.55)	<0.001*	1.80 (1.10–2.94)	0.019*
Comorbidity				
No	1.00		1.00	
Yes	3.97 (2.64–5.97)	<0.001*	2.26 (1.20–4.27)	0.012*
Adverse events				
No	1.00		1.00	
Yes	1.45 (1.04–2.02)	0.028*	1.09 (0.68–1.77)	0.720
Regimen type at baseline				
Standardised	1.00			
Modified individualised	1.55 (1.10–2.19)	0.013*		

*OR* Odds ratio, 95 % CI (Confidence interval)

^a^Unadjusted model

^b^Adjusted for BMI, haemoglobin, CD4, and other opportunistic infections

*Significant *p* <0.05

### Predictors of treatment failure

Patients with severe anaemia had nearly 4.7 times higher rate of failure compared with patients with normal haemoglobin level (OR 4.72, 95 % CI 1.47–15.08, *p* = 0.009). The presence of other co-morbidities was associated with a 2.4 times higher rate of failure compared with those without other co-morbidities (OR 2.39, 95 % CI 1.05–5.43, *p* = 0.039). Patients on modified individualised regimen at baseline had a 2 times higher rate of failure compared with patients on standardized regimen at baseline (OR 2.15 95 % CI 0.98–4.71, *p* = 0.056), as observed in Table 5.

**Table 5:** Predictors of treatment failure in HIV-positive adults on MDR-TB treatment at Sizwe Tropical Disease Hospital, Johannesburg

	^a^Univariable Analysis		^b^Multivariable Analysis	
Factor	IRR (95 % CI)	*p*-value	IRR (95 % CI)	*p*-value
ART regimen				
After	1.00		1.00	
Prior	1.01 (0.47–2.15)	0.988	0.80 (0.35–1.84)	0.606
Age groups				
18–45	1.00			
46–76	0.79 (0.27–2.28)	0.662		
BMI	0.97 (0.87–1.07)	0.547	1.01 (0.94–1.09)	0.766
Haemoglobin (g/dl)				
Severe Anaemia (<7.0)	2.69 (0.78–9.30)	0.117	4.72 (1.47–15.08)	0.009*
Moderate Anaemia (7.0–9.99)	1.09 (0.46–2.57)	0.839	0.97 (0.40–2.34)	0.944
Mild Anaemia (10–10.99)	0.43 (0.10–1.87)	0.260	0.25 (0.03–1.82)	0.172
Normal (≥11)	1.00		1.00	
Infiltrative changes				
No	1.00			
Yes	1.01 (0.14–7.47)	0.990		
Cavitary changes				
No	1.00		1.00	
Yes	1.81 (0.85–3.87)	0.125	2.14 (0.96–4.78)	0.064
Fibrotic changes				
No	1.00			
Yes	1.36 (0.41–4.50)	0.620		
Comorbidity				
No	1.00		1.00	
Yes	1.79 (0.72–4.41)	0.207	2.39 (1.05–5.43)	0.039*
Adverse events				
No	1.00		1.00	
Yes	0.54 (0.24–1.23)	0.142	0.48 (0.21–1.09)	0.080
Regimen type at baseline				
Standardised	1.00		1.00	
Modified individualised	2.48 (1.18–5.20)	0.016*	2.15 (0.98–4.71)	0.056

*IRR* Incidence rate ratio

^a^Unadjusted models

^b^Adjusted for Co-trimoxazole prophylaxis, BMI and categories of MDR-TB

*Significant *p* <0.05

## Discussion

The study aimed at ascertaining the clinical characteristics and outcomes of MDR-TB in HIV co-infected patients based on timing of ART initiation, as well as determining the predictors of mortality, and treatment failure. This study revealed a higher proportion of new MDR-TB cases and the presence of other non-TB opportunistic infections among patients who initiated ART after commencing MDR-TB. These findings point to the importance of early initiation of ARVs and its protective benefits for HIV positive patients. On the whole, outcomes of MDR-TB did not differ significantly between patients who started ART before or after initiation of MDR-TB except for mortality which was higher among patients who commenced ART before initiating MDR-TB treatment. Risk factors for mortality were the use of ART before the commencement of MDR-TB, being severely underweight and underweight, cavitary lesions on baseline chest x-ray, the presence of other opportunistic infections and other co-morbidities. Severe anaemia at baseline, and the presence of other co-morbidities were associated with higher rates of treatment failure.

### Predictors of mortality

Antiretroviral therapy use prior to MDR-TB treatment initiation was significantly associated with higher odds of mortality compared with ART initiation after commencement of MDR-TB treatment. This finding is surprising and has not been reported before by other studies. It should be assumed that patients should have benefited from being on ARVs before MDR-TB treatment. Based on this data, a greater percentage of patients who commenced ART before MDR-TB treatment initiation were severely underweight. They also had more extra-pulmonary TB, adverse events, and modified regimen based on patients’ history at baseline compared with patients who started ART after initiation of MDR-TB treatment at baseline. A lack of effect of ART from poor adherence, and treatment failure may have been responsible for this trend. The availability of viral load data at baseline would have further buttressed this fact, but this was not done routinely during the study period.

Prior to 2010, MDR-TB patients were initiated on ARTs if their CD4 cell count was <350 cell/mm^3^ based on the WHO guidelines [13]. Thereafter, ART was encouraged to be initiated within 6–8 weeks of starting MDR-TB treatment irrespective of CD4 count [15]; hence prior to 2010, patients with higher CD4 cell count were monitored until their CD4 counts dropped to <350 cells/mm^3^. This trend also explains the presence of more EPTB and the slightly lower baseline weight and the mortality seen in this group. Following prior initiation of ART, their CD4 cell counts may have improved such that they appeared similar CD4 cell count as with patients who started on ART after initiating MDR-TB treatment at baseline, as noted in our study.

This result poses questions on the issue of adherence to ART for patients who were already on ART prior to MDR-TB treatment in a country like South Africa with majority of HIV positive patients on ARVs. Additionally, drug-drug interactions between the MDR-TB and ARVs pills may have resulted in the high mortality in this study. IRIS was considered as the most potential explanation for this finding simply due to the fact that when analysis was restricted to patients who were on ART within six months as opposed to 3 months prior to commencement of MDR-TB treatment compared with those who started on ART after initiating MDR-TB treatment, no statistically significant relationship was observed with prior ART initiation and mortality. This further affirms the rigorous monitoring and management of IRIS by attending clinicians. Clinicians should not undervalue the role of adherence to ART treatment in patients who are already on ART before starting MDR-TB treatment. Appropriate management of already existing adverse events, opportunistic infections and co-morbidities in these patients are important to maximise the protective benefits of being on ART before initiating MDR-TB treatment, and on the whole achieve better treatment outcomes.

This study found that being severely underweight and underweight were risk factors of mortality. These findings are similar to previous studies [9, 12, 17–19]. HIV and TB are linked to malnutrition [18]; and wasting syndrome is a hallmark of severity of TB and HIV infection, therefore its strong association as a predictor of mortality irrespective of patients’ ART status is not surprising. A Cavitary lesion seen on chest x-ray at baseline was a risk factor of mortality. There have been conflicting studies on the role of cavitation on chest x-ray as a risk factor of poor outcomes. Some studies have linked the presence of cavitation on chest x-rays with longer culture conversion time; a surrogate marker for poor outcomes [20–22]. Others have found no statistically significant relationship between cavitation and poor outcomes [17, 23]. In a recent study done by Brust JC et al. in South Africa, its failure of finding a significant relationship between cavitation and longer time to sputum conversion may have been due to the limited sample size of 56 patients [23].

The presence of other non-TB opportunistic infections was a significant predictor of mortality. This finding has not been reported before by other studies beyond the descriptive level [9]. There is a higher risk of mortality and development of other opportunistic infections in HIV-TB co-infected patients [24, 25]. The role of other non-TB opportunistic infections is important in the clinical and prognostic staging of HIV positive patients. Serious opportunistic infections like Kaposi sarcoma, cervical cancer, Cytomegalovirus (CMV) infections, HIV-associated nephropathy (HIVAN), etc. are already indicators of the depressed immunity with an increase likelihood of mortality masking the importance of timing of ART initiation in these patients.

In this study the presence of co-morbidities was a significant risk factor of mortality. This result was reported in a recent study in the United Kingdom and Peru [26, 27]. However, the proportion of co-morbidities like diabetes, chronic kidney disease, and chronic liver disease was higher than that reported in the United Kingdom. There is an established “bidirectional synergistic” relationship between TB-HIV co-infection and the development of co-morbidities like DM [28–30]. Treatment of HIV with ART especially with the protease inhibitors is associated with hyperglycaemia, insulin resistance, dyslipidaemia [31]; not only leading to the development and complication of DM, but cardio-vascular, cerebro-vascular, hepatic and renal problems. Most studies done on the role of certain co-morbidities on TB mortality focus on single disease entity like DM; with positive [32], and negative [17] results. This study is the first in South Africa to incorporate the presence of other non-communicable co-morbidities apart from DM to assess its impact on mortality in MDR-TB HIV co-infected patients, especially with the emerging link of infectious and non-communicable diseases.

### Predictors of treatment failure

Severe anaemia (haemoglobin <7.0 mg/dl) at baseline predicted treatment failure. The causes of anaemia in MDR-TB HIV co-infected patients are multi-factorial; resulting from the HIV/mycobacterial infection and treatment with both anti-TB and ARV medications. This finding is consistent with that of Mitnick C, et al. where low haematocrit was associated with about 4 times increase in the hazard of failure or death compared with normal haematocrit [18].

This study found that the presence of other co-morbidities was associated with a higher rate of treatment failure. The co-existence of other communicable and chronic non-communicable medical conditions such as DM, heart failure, cerebrovascular diseases, renal diseases, etc. increases the pill burden, adverse events and drug-drug interactions for HIV co-infected MDR-TB treatment. These patients require regimen modifications based on their clinical state, and hence may affect adherence to both ART and MDR-TB treatment, and lead to treatment failure. DM has been associated with MDR-TB [33] and PTB [34] treatment failure; however these studies did not consider the effect of other co-morbidities.

Modified individualised regimen at baseline for patients based on co-morbidities and adverse-events was significantly associated with treatment failure. The treatment of MDR-TB utilizes at least five medications at baseline and the WHO advocates the use of DST results to guide treatment regimen. In the practical clinical setting the presence of severe adverse-events, co-morbidities, and pre-XDR strains guide the clinicians on what regimen to use in initiating MDR-TB treatment regimen not just the DST result. Therefore for some of these patients certain drugs were delayed or added (in cases of pre-XDR strains) at baseline pending improvement in clinical condition. For example, Kanamycin/Amikacin were omitted and substituted with other drugs when there was renal toxicity. Terizidone was substituted if there was a serious psychiatric side effect. In cases of pre-XDR TB, the deviation was solely based on available antibiogram showing resistance to the injectable or Fluroquinolone. These patients termed as those on “modified individualized regimen” in this study to reflect the challenges clinicians face when initiating MDR-TB treatment using DST results bearing in mind patient centred care. This finding supports the result by Leimane V et al. which noticed that patients on ≤ 5 drugs for 3 months or more were more likely to fail or die [19].

### Limitations and strengths

This study depended on already collected data from medical records, hence may not have measured all the possible confounders. Viral load for patients would have been a true test of adherence especially for patients on ART prior to initiating MDR-TB treatment, thereby helping in the explanation of the relevant findings. However, viral load could not be used as the definition of virological suppression varied over the years according to the NHLS definitions. Additionally only few data were collected for this variable because during this period viral load was expensive and could not be afforded for all patients at baseline except where patients were suspected to have had immunological or clinical failure.

In this study, timing of ART was stratified as before or after commencement of MDR-TB treatment and this may not have allowed proper assessment of the true effect of duration of ART on the outcomes. Information on baseline ART regimen were not collected, which would have been a pointer to drug resistance and virological failure and may have possibly helped in explaining the role of timing of ART on mortality in terms of adverse-events and toxicity of individual drugs. Coding co-morbidities as a yes or no variable instead of looking at individual diseases may have overestimated the effect of such variable on the outcomes in patients who had one or two co-morbidities compared to those who had a combination of highly fatal co-morbidities. Findings of this study may not be generalizable to regions with low ART coverage.

This is one of the largest cohorts of MDR-TB HIV co-infected patients, allowing reasonable power to detect a difference between the two exposure groups in relation to the major outcomes and other risk factors for mortality, and failure. The objective measurements of the outcomes minimised misclassification bias as majority of patients were confirmed as cured or failed treatment based on TB culture results from NHLS and extensive follow-up. Mortality was also confirmed in the hospital and for patients who died at home, regular tracing and reminders for visits helped in ascertaining home-related mortality. Certain variables like weight, height, CD4 count, haemoglobin, and chest x-rays were collected within specified periods to prevent disparate exposure duration hence, preventing over or under-estimation of the effect size. Information on certain exposures like other opportunistic infections, co-morbidity and adverse events were collected and this may have helped explain the predictors of mortality, and failure. The standard practice of ascertaining patients’ ART status at baseline and documentation of definite start dates by physicians at Sizwe Hospital reduced the likelihood of misclassification bias based on ART status.

## Conclusions

This study provides new information while also reinforcing old knowledge to clinicians and public health practitioners for the identification of TB populations at higher risk of death, and treatment failure. Mortality was higher in patients on ART prior to start of MDR-TB treatment compared with those initiated on ART after commencing MDR-TB treatment. Strict adherence to ART and monitoring for drug-drug interactions, management of adverse-events and existing co-morbidities in HIV positive patients irrespective of their TB status is pivotal to achieving favourable outcomes. The study results also places an emphasis on the role of Public health practitioners to intensify intervention programmes targeted at early screening and identification of MDR-TB, treatment initiation, increasing adherence and drug monitoring for HIV infected patients.
